# The Impact of Maxillary Sinus Pneumatization on the Quality of the Alveolar Bone in Dentated and Edentulous Patients: A Cone-Beam Computed Tomography Radiographic Analysis

**DOI:** 10.7759/cureus.46005

**Published:** 2023-09-26

**Authors:** Shadia A Elsayed, Muath S Alassaf, Mohamed O Elboraey, Lamis L Mohamado, Dalia A Huwaykim, Alwaleed K Albouq, Maher O Shahada

**Affiliations:** 1 Oral and Maxillofacial Surgery, Taibah University, Madinah, SAU; 2 Oral and Maxillofacial Surgery, Al-Azhar University, Cairo, EGY; 3 Orthodontics and Dentofacial Orthopedics, Taibah University, Madinah, SAU; 4 Oral Medicine, Periodontology, Oral Diagnosis and Radiology, Tanta University, Tanta, EGY; 5 Periodontology, Taibah University, Madinah, SAU; 6 Dental Education, Taibah University, Madinah, SAU

**Keywords:** maxillary sinus lift, teeth loss, sinus pneumatization, dental implant, bone density, maxillary sinus

## Abstract

Background

This study aimed to describe the morphometric differences of maxillary alveolar bone’s height, width, and densitometric differences in the posterior region where maxillary sinus pneumatization occurred.

Methodology

A cross-sectional, cone-beam computed tomography (CBCT) study used 123 CBCT images as a non-randomized convenient sample of sinus pneumatized cases. Bone height, bone width (in mm), and average density of the remaining ridge of all patients were used as study variables. Analysis of the qualitative variables were as frequency and percentages. Parametric Student’s t-test and non-parametric chi-squared tests were used to compare the groups. The significance level was set at a p-value ≤0.05.

Results

The sample included CBCT radiographs for patients who had a mean age of 42.79 ± 12.32 with males constituting 69 (56.1%) of the patients. There was no gender difference between the present and missing teeth at the measured sites of the first premolar, second premolar, first molar, and second molar (p > 0.05). The mean measurements of height and average bone density were significantly higher in the dentate sites; however, the mean width was higher in the edentulous sites (p = 0.001).

Conclusions

Average bone height and density were significantly decreased at the edentulous sites of sinus pneumatized cases than the dentate sites with no gender difference.

## Introduction

Sinus pneumatization is a physiological process in which the volume of the paranasal sinuses increases over time [1,2]. Tooth loss is thought to cause maxillary sinus pneumatization (MSP), which can lead to a union between the sinus floor and the alveolar crest in severe cases. According to certain studies comparing pre- and post-extraction radiographs, MSP may develop following posterior tooth extraction [3]. In clinical practice, ridge resorption in the coronal area of the extraction socket might reduce the available bone height for future implant insertion. The mechanical strength of the bone tissue surrounding the extraction site may also be reduced as a result of MSP [4,5].

The rate of alveolar bone resorption varied considerably depending on the individual and tooth position [2]. Several factors can influence this, including infection, previous periodontal disease, the severity of a traumatic injury, and the quantity and thickness of the bone socket walls [6]. Accurately measuring alveolar bone height and thickness in implant treatment is critical. For example, reduced alveolar bone height and thickness have been related to labial flaring, extrusion, rotation, spacing, and drifting of the teeth, all of which can lead to a complex malocclusion that requires interdisciplinary treatment. The height, thickness, volume, and quality of alveolar bone all play a role in determining the ideal location for dental implantation and its prognosis [7,8].

Several studies have attempted to classify the morphological changes in maxillary bony anatomy following tooth loss, which is essential information for pre-prosthetic surgery and treatment planning [9]. Considering the importance of the maxillary sinus for implant placement in the upper posterior region, an accurate diagnosis and a better understanding of bone remodeling in that area could be highly beneficial for treatment planning. A secondary analysis was performed to determine whether maxillary sinus lift is necessary and to identify anatomical factors that may direct sinus floor pneumatization [10].

Therefore, the purpose of this study was to quantitatively measure and report bone height, width, and density of the maxillary alveolar bone in the posterior region, which had MSP among patients of Taibah University, College of Dentistry and Dental Hospital, Al-Madinah Al-Munawwarah, Saudi Arabia. To answer the question, to what extent does sinus pneumatization affect the patient’s bone height and density in the posterior maxillary areas?

The hypothesis is that sinus pneumatization among Taibah patients decreases the morphometric measurements of the remaining alveolar ridge (density, height, width) and gender may have an effect on these changes.

## Materials and methods

Study design

This was a cross-sectional radiographic study using cone-beam computed tomography (CBCT) images to assess bone height, width, and density in areas of sinus pneumatization in posterior maxillary areas. CBCT scans were done in the College of Dentistry at Taibah University. Between 2019 and 2023, 1,000 CBCT scans performed at Taibah University’s College of Dentistry were reviewed to identify cases with sinus pneumatization in both dentate and non-dentate areas and no odontogenic causes of crestal bone loss. Scans performed between 2019 and 2023 were screened to include cases that met the eligibility criteria. The sample size was determined using parameters that closely matched the Saudi population: 80% power, a 95% confidence interval (CI), and a 0.05 margin of error.

Patient eligibility criteria

Patients who had an extraction of at least one maxillary posterior tooth on one side with an intact contralateral side were included. In this study, we targeted the age group of 18-70 years. Patients with crestal bone loss were excluded from the study. A horizontal line was drawn from the alveolar bone crest of the mesial and distal neighboring teeth to the edentulous area. Then, the distance from the alveolar bone crest to the horizontal line (distance C) was measured. If distance C was more than 3 mm, the case was excluded from the study; this was done to ensure that the alveolar bone height defect was only due to sinus pneumatization. The cutoff thresholds were chosen based on Saumya et al.’s earlier work, which described the amount of crestal bone height and width changes following extraction [11]. For edentulous areas, cases with bone height less than 10 mm without crestal bone loss of more than 3 mm were considered pneumatized. The case was considered pneumatized for the dentated side if the bone height was less than 10 mm. These cutoff points were determined using data from a recent study by Elsayed et al., who examined MSP measurements alongside potential correlations in Al-Madinah Al-Munawwarah [12].

Study variables and data collection technique

Two investigators screened 2,300 scans to include those that fit the eligibility criteria. The sample of 123 CBCT images was used as a non-randomized convenient sample. Age, names, gender, side, bone height (mm), and density of the remaining ridge of all patients were the study variables. The CBCT machine at CDTU (Carestream CS 9300) was used to obtain all scans in this study. Parameters of the machine were set to 90 kVp and 4 mA and eight seconds of acquisition time.

**Figure 1:**
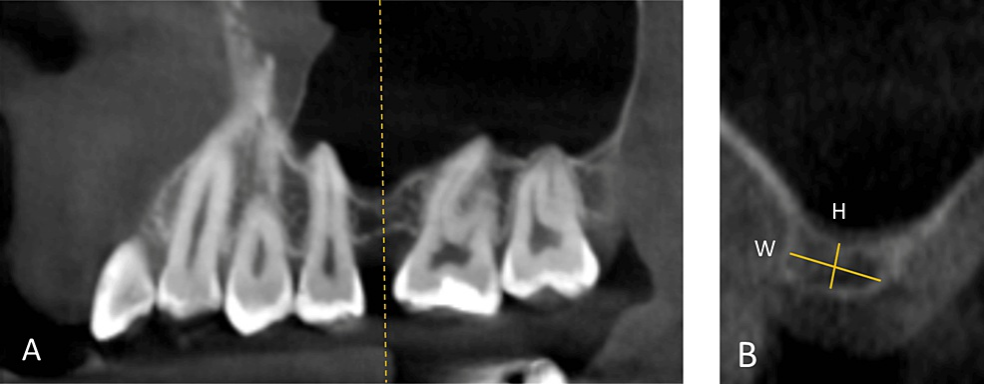
Measurement method in edentulous cases. A: Sagittal cut used to orient the coronal cut. B: Coronal cut showing the height (H) and width (W) measurements.

**Figure 2:**
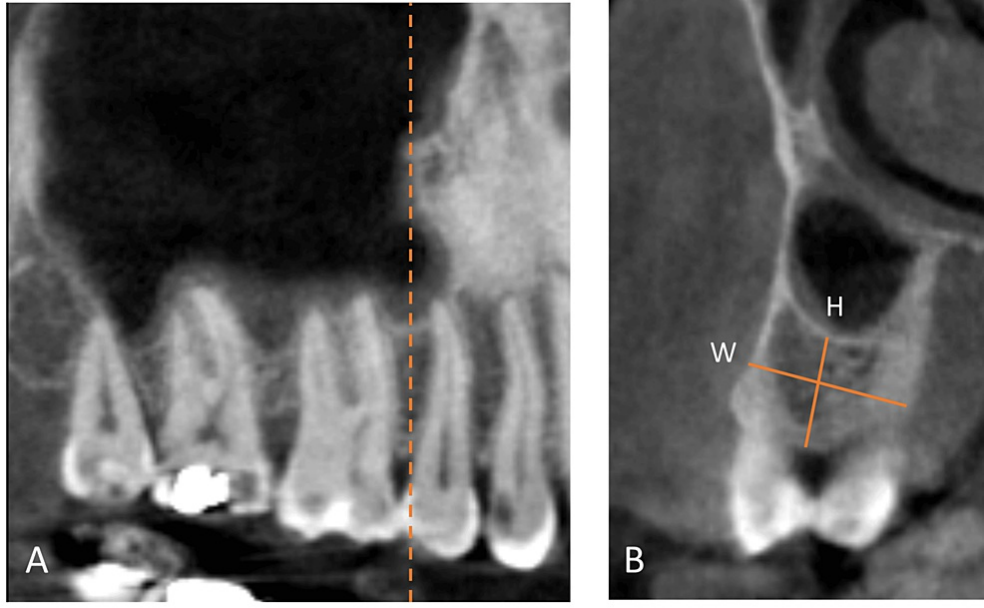
Measurement method on the dentated side. A: Sagittal cut used to orient the coronal cut in an area on the alveolar bone. B: Coronal cut showing the height (H) and width (W) measurements.

The coronal cut of the CBCT image was oriented at mid-distance between the adjacent alveolar crests and used to measure the bone height from the crestal ridge to the maxillary sinus floor and the alveolar ridge’s bone width, as shown in Figure 1 for the edentulous side and Figure 2 for the dentated side.

Bone density was measured for both edentulous and dentate patients using Blue Sky Bio software version 4.7 to measure the density, height, and width of the alveolar ridge. After being trained and calibrated, two observers were given the task of performing the measurements. This involved checking the measurement uniformity across five CBCTs. The identical measures for each CBCT were obtained. Points used to measure the bone density were distributed to cover all the remaining alveolar ridges in edentulous patients, while for dentated patients, the points were distributed at the interdental and inter-radicular bone to avoid the density measurement for the present teeth roots. The main bone density was calculated from the average of the measured points. In case of disagreement, the average of the two values was used. Intra and interexaminer testing was conducted.

Ethical considerations

Ethical approval for this study was obtained from the Taibah University College of Dentistry Research Ethics Committee, and a waiver of informed consent was approved (reference number: TUCDREC/04042022/SAElsayed).

Data analysis

SPSS version 16 (SPSS Inc., Chicago, IL, USA) was used to analyze the data from an Excel sheet. The sample characteristics were reported using descriptive analysis. Continuous variables were presented as mean with standard deviations if the data coincided with normality distribution (Kolmogorov-Smirnov, p > 0.05). Qualitative variables were analyzed as frequency and percentages. In comparing groups, appropriate parametric Student’s t-test and non-parametric chi-square test were used. The significance level was set at a p-value ≤0.05.

## Results

The gathered data were analyzed using SPSS software version 25. The sample consisted of 123 radiographs taken from patients with a mean age of 42.79 ± 12.32, of whom 69 (56.1%) were males.

Table 1 shows descriptive information on the frequency and percentage of gender and the number of edentulous sites at each first and second premolar and first and second molar on both the right and left sides.

**Table 1:** The demographic data distribution of the patients (n = 123).

Study variable	Variable type	Frequency (%)
Gender	Male	69 (56.1%)
	Female	54 (43.9%)
Side	Right	66 (53.7%)
	Left	57 (46.3%)

The distribution of the missing and present teeth in relation to the maxillary sinus, together with morphometric details, is shown in Table 2. The independent-sample t-test was used to compare the means of the dependent variables, which included height, width, and density values between the two independent edentulous and dentate sites of each region, which revealed that there was a significant difference in mean height, width, and density between edentulous and non-edentulous sites (Table 3).

**Table 2:** Distribution of the missing and present teeth in relation to the maxillary sinus, together with morphometric details (n = 123).

Site, n (%)	Edentulous	Dentated
First premolar	26 (21.1%)	97 (78.9%)
Second premolar	32 (26%)	91 (74%)
First molar	50 (40.7%)	73 (59.3%)
Second molar	28 (22.8%)	95 (77.2%)
Height	11.1 ± 5.2	14 ± 4.3
Width	12.1 ± 28.9	11.9 ± 2.78
Average density	676.3 ± 288.8	849.2 ± 343.1

**Table 3:** Morphometric study variables measured in both edentulous and dentate areas (n = 123).

Measurement site	Mean	Standard deviation	Standard error mean	P-value
Edentulous height	11.06	5.28	0.48	0.001
Dentulous height	14.01	4.31	0.39	
Edentulous width	12.13	28.85	2.6	0.001
Dentulous width	11.89	2.78	0.25	
Edentulous average density	676.34	288.9	26.05	0.001
Dentulous average density	849.22	343.9	30.93	

T-test was used to compare the difference between the edentulous and dentate sites in terms of the mean of alveolar ridge height and width, as well as measured density, and revealed statistically significant differences between dentated and edentulous ridge (p = 0.001), with the mean height and bone density being higher in the dentate sites. Still, the mean width was higher in the edentulous sites (Table 3).

Chi-square analysis of the significance difference between the various measurements and the correlation with gender and side were analyzed, and the findings indicated that there was no significant difference regarding edentulous or dentate status at any of the sites under investigation regarding age and gender, as shown in Table 4 (p = 0.135).

**Table 4:** Cross-tabulation of gender versus comparison of different sites between edentulous state and dentate state (n = 123).

Gender	First premolar		Second premolar		First molar		Second molar	
	Present	Missing	Present	Missing	Present	Missing	Present	Missing
Male	53 (43.1%)	16 (13.0%)	51 (41.5%)	18 (14.6%)	40 (32.5%)	29 (23.6%)	50 (40.7%)	19 (15.4%)
Female	44 (35.8%)	10 (8.1%)	40 (32.5%)	14 (11.4%)	33 (26.8%)	21 (17.1%)	45 (36.6%)	9 (7.3%)
P-value	0.529		0.984		0.725		0.154	

The bivariate correlational test found that the density was statistically positively connected with the ridge height and that an increase in height was correlated with an increase in density (p = 0.009), while the width of the ridge was not correlated with either the height or the density of the ridge (p ≥ 0.05) (Table 5).

**Table 5:** Correlations analysis between different study variables (n = 123). **: Correlation is significant at the 0.01 level (two-tailed).

Variables	Test	Edentulous height	Edentulous width	Edentulous average density
Edentulous height	Pearson correlation	-	0.014	0.234
	Significance (two-tailed)	-	0.874	0.009**
Edentulous width	Pearson correlation	0.014	-	-0.068
	Significance (two-tailed)	0.874	-	0.453
Edentulous density	Pearson correlation	0.234	-0.068	-
	Significance (two-tailed)	0.009**	0.453	-

## Discussion

The maxillary sinus continues to pneumatize as the alveolar ridge develops until the alveolar ridge is completed and the third molar erupts at age 20. Except for tooth loss, there are no clear reasons for sinus pneumatization following full alveolar development. As there are no data or studies available regarding the assessment of the degree of alveolar ridge remaining pattern and the alveolar bone changes with clinical implications for oral and maxillofacial surgeons and implantologists in the Al-Madinah Al-Munawwarah, Saudi Arabia population, this study was performed to evaluate alveolar ridge dimensions and morphometric changes in a group of sinus pneumatized Al-Madinah Al- Munawwarah patient population.

Previous studies have demonstrated that the structural alterations brought on by a single or a series of tooth extractions can significantly alter the edentulous ridge’s profile. However, a prior study in a different section of the Saudi population revealed that a high percentage of young adult dentate individuals experienced MSP [12,13]. Numerous maxillary edentulous cases necessitate extra surgical operations, including sinus lifts and bone grafts, to insert implants due to significant bone resorption and MSP [14,15].

The present comparison of the morphometric changes revealed that both the mean of ridge height and density had significantly decreased at the edentulous areas of the measurement sites despite these deficiencies still falling within accepted measurements for ridge implantation. This result is in accordance with the findings of Farina et al., who compared the alveolar ridge dimensions between edentulous sites and contralateral dentate sites of maxillary posterior sextants in the same individuals [14]. The difference between alveolar bone density in edentulous and dentate patients was not due to present teeth roots, as it was already excluded during the density measurements. There are many explanations for the results; the density changes may be due to mechanical interaction between the present teeth and the alveolar bone during function and its impact on the present osteoblasts for more bone mineralization.

Additionally, the statistical comparison and study findings showed that the average ridge morphometric measurements are within normal limits, and, in most cases, there is no need for additional surgical work for sinus augmentation in our community’s population. This agrees with the measurements of the Wagner study [9]. Short implants or closed sinus lift procedures might be used to make up for these minor deficiencies. This was in accordance with the recommendation of Tolstunov et al., who also found that ridge volume was sufficient in most cases with sinus pneumatization based on their CBCT study [15].

In addition, this study showed that gender has no impact on the maxillary ridge configuration’s morphometric measures changes. However, gender was statistically significant in other studies that examined changes in mandibular height [16].

According to Lim et al., the greatest sinus pneumatization occurred following the second molar extraction [4]. Others, such as Levi et al., recommend socket preservation after extraction to decrease the sinus pneumatization in the posterior maxilla [6]. In this study, the first molar had the most frequent sinus pneumatization. The conserved ridge region was at the first premolar, which is consistent with many investigations, which could be because it erupted early and was most vulnerable to decay and extraction [17-20].

Preoperative planning should include periapical, panoramic, and conventional CT scans. In many clinical settings, three-dimensional imaging is more effective than two-dimensional imaging in overcoming its limitations [21]. Using three-dimensional imaging instead of two-dimensional imaging can be helpful to overcome restrictions [22].

CBCT is the gold standard technique in dentistry for diagnosis and treatment planning, with several benefits, including lower radiation doses, high accuracy, and three-dimensional measurements [23]. CBCT is a supplementary diagnostic method that produces 3D pictures [24]. It significantly lowers anatomical structural overlap, allowing for a more accurate assessment of the alveolar bone and maxillary sinus floor condition, especially on the palatal floor [2,10].

Typically, morphometric studies are significant, and presentation of the morphometric dimensions of the alveolar ridges in individuals who had sinus pneumatization may provide the knowledge necessary to guide surgical maxillary implantation in our population and its related structures [25]. Therefore, to answer the question posed by the study “To what extent does sinus pneumatization affect the patient’s alveolar bone height and density in the posterior maxillary areas?” even with the presence of sinus pneumatization, both dentated and edentulous posterior alveolar ridge dimensional changes, and density present within the normal range.

In this research, the hypothesis was that sinus pneumatization of Taibah patients decreases the morphometric measurements of the remaining alveolar ridge (density, height, width) and gender may affect these changes. The hypothesis is now partially accepted in which the results demonstrated a significant decrease in both alveolar height and density due to MSP, regardless of gender.

Limitations

Although significant outcomes were obtained, this study needed to be more extensive in the aspect of sample size, as a larger sample size would have revealed more findings and correlations. In addition, the scans used were from one institution; a multicenter study from different regions may result in more generalizable knowledge. Age stratification was not done in the statistical analysis, which is considered a limitation of the current work. Future studies are needed to compare alveolar bone density in edentulous patients with and without sinus pneumatization to answer whether sinus pneumatization causes alveolar bone compression reflected by increased bone density in pneumatized patients.

## Conclusions

This study explored the effects of sinus pneumatization on alveolar ridge metrics and showed that alveolar height and density significantly decreased with MSP. Gender had no significant impact. The most common site of sinus pneumatization was the first molar. CBCT is crucial for accurate assessments and planning for implant placement.
